# KIAA1363—A Multifunctional Enzyme in Xenobiotic Detoxification and Lipid Ester Hydrolysis

**DOI:** 10.3390/metabo12060516

**Published:** 2022-06-02

**Authors:** Carina Wagner, Victoria Hois, Ulrike Taschler, Michael Schupp, Achim Lass

**Affiliations:** 1 Institute of Molecular Biosciences, NAWI Graz, University of Graz, 8010 Graz, Austria; carina.wagner@uni-graz.at (C.W.); ulrike.taschler@uni-graz.at (U.T.); 2 Division of Endocrinology and Diabetology, Medical University of Graz, 8036 Graz, Austria; victoria.hois@medunigraz.at; 3 Cardiovascular Metabolic Renal (CMR)—Research Center, Institute of Pharmacology, Charité—Universitätsmedizin Berlin, Corporate Member of Freie Universität Berlin and Humboldt-Universität zu Berlin, 10115 Berlin, Germany; michael.schupp@charite.de; 4 BioTechMed-Graz, 8010 Graz, Austria; 5 Field of Excellence BioHealth, 8010 Graz, Austria

**Keywords:** KIAA1363, neutral cholesterol ester hydrolase 1, arylacetamide deacetylase-like 1, lipid metabolism, xenobiotics

## Abstract

KIAA1363, annotated as neutral cholesterol ester hydrolase 1 (NCEH1), is a member of the arylacetamide deacetylase (AADAC) protein family. The name-giving enzyme, AADAC, is known to hydrolyze amide and ester bonds of a number of xenobiotic substances, as well as clinical drugs and of endogenous lipid substrates such as diglycerides, respectively. Similarly, KIAA1363, annotated as the first AADAC-like protein, exhibits enzymatic activities for a diverse substrate range including the xenobiotic insecticide chlorpyrifos oxon and endogenous substrates, acetyl monoalkylglycerol ether, cholesterol ester, and retinyl ester. Two independent knockout mouse models have been generated and characterized. However, apart from reduced acetyl monoalkylglycerol ether and cholesterol ester hydrolase activity in specific tissues and cell types, no gross-phenotype has been reported. This raises the question of its physiological role and whether it functions as drug detoxifying enzyme and/or as hydrolase/lipase of endogenous substrates. This review delineates the current knowledge about the structure, function and of the physiological role of KIAA1363, as evident from the phenotypical changes inflicted by pharmacological inhibition or by silencing as well as knockout of KIAA1363 gene expression in cells, as well as mouse models, respectively.

## 1. Introduction

The arylacetamide deacetylase (AADAC) protein family name giving protein AADAC is a type II membrane glycoprotein, facing with its active side to the lumen of the endoplasmic reticulum (ER) [1]. In human tissues, it is highly expressed in the liver and intestine [1,2,3,4,5]. Its expression follows a diurnal rhythm, is highest in the post-absorptive (fasted) state, and is upregulated by peroxisome proliferator-activated receptor-α (PPARα) [3]. AADAC shares some sequence homology and substrate specificity with hormone-sensitive lipase (HSL, a well-studied multifunctional neutral lipid ester hydrolase) [2,3]. AADAC substrates include neutral lipids such as diglycerides, but also several xenobiotics, including a number of clinical drugs, such as the antiandrogen drug flutamide, the analgesic antipyretic drug phenacetin, and the antituberculosis drug rifamycin [1,4,5,6,7]. Evidence that AADAC is involved in lipid metabolism has been derived from cell experiments: the expression of AADAC in rat hepatoma cells decreased intracellular triglyceride stores that was accompanied by increased fatty acid oxidation and impaired secretion of lipoproteins [1]. Interestingly, in the in vitro activity lipase assays using endogenous ER substrates, membrane fractions of hepatoma cells stably expressing AADAC showed depletion of diglyceride but not triglyceride or cholesterol ester [1], suggesting that AADAC has substrate preference for diglyceride as demonstrated for HSL [8].

The hydrolytic activities and functional role of AADAC suggest that other members of this protein family may exhibit similar features. The AADAC family comprises five members, including AADAC and four AADAC-like (AADACL1-4) proteins. Except for AADACL1, more commonly known as KIAA1363, all other members of the AADACL protein family, AADACL2, AADACL3, and AADACL4, have so far been only poorly investigated and no functional roles can be concluded. A number of studies have identified hydrolytic activities and derived functional roles for KIAA1363. In the next sections, we explore the current knowledge about the structure, expression profile, and enzymatic activities of KIAA1363 and discuss its functional role.

## 2. Gene Organization and mRNA Variants

**Figure 1:**
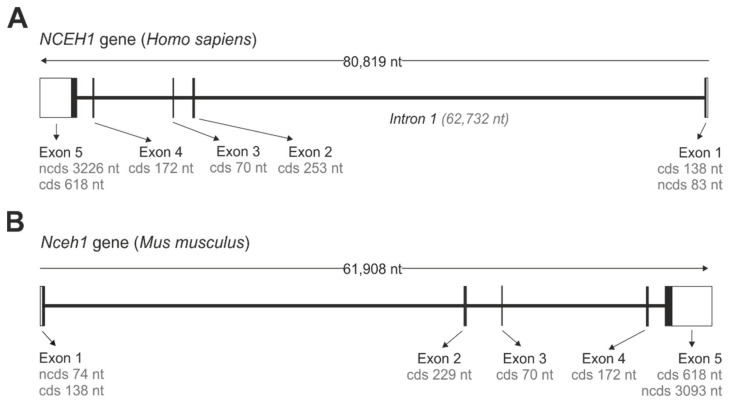
Gene organization of *NCEH1/Nceh1*. Gene organization of the human *NCEH1* (**A**) and murine *Nceh1* (**B**). Open boxes (white) represent non-coding 5′ and 3′ nucleotide sequences (ncds). Closed boxes (black) represent exons that are separated by intron stretches, represented by interconnecting lines. Lengths of non-coding and coding sequences (cds) are indicated.

The gene encoding for murine and human KIAA1363 is annotated as *neutral cholesterol ester hydrolase 1* (*Nceh1/NCEH1)*. Both the murine and human *Nceh1/NCEH1* genes are located on chromosome 3 in the chromosomal regions 3qA3 and 3q26.31, respectively, and share common structural features. They consist of five protein-encoding exons, spanning between 61 and 81 kb (Figure 1). Interestingly, the open reading frame of the murine gene is encoded on the plus strand, while that of the human gene is encoded on the minus strand (Figure 1). From all exons, exon 5 contains the largest protein coding sequence of 618 nt, while of all introns intron 1 is by far the largest and spans 62,732 nt in the human *NCEH1* gene.

**Figure 2:**
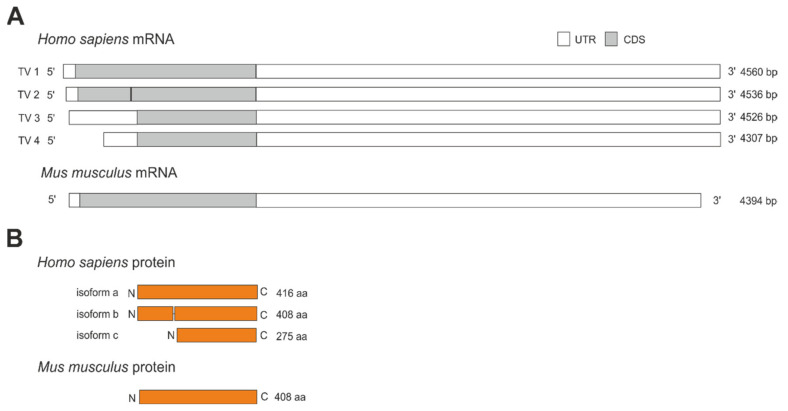
Transcript variants and protein isoforms of human and murine *Nceh1*. (**A**) The human *NCEH1* gene transcribes into 4 different transcript variants (TV) generated by alternative splicing or translation start codon usage ranging from 4307 to 4560 bp. Transcript variants vary in the 5′ and 3′ untranslated region (UTR) and coding sequence (CDS). The murine *Nceh1* gene transcribes into a single 4394 bp long mRNA. (**B**) Human protein isoform A consists of 416 amino acids (aa). Isoform B is lacking 8 aa (position 123–130), leading to a shorter 408 aa protein. Isoform C is lacking 141 aa at the N-terminus, leading to the shortest protein consisting of 275 aa. The murine *Nceh1* gene encodes for a single protein isoform consisting of 408 aa.

The human *NCEH1* gene transcribes into four mRNA variants (Figure 2A). These variants are produced by alternative splicing and translate into three different protein isoforms (Figure 2B). Transcript variant 1 encodes for the longest isoform A consisting of 416 amino acids with a calculated molecular weight of 46,627 Da. Transcript variant 2 is generated by the usage of an alternative in-frame splice variant in the 5′ region, resulting in a shorter isoform B lacking eight amino acids from position 123 to 130, leading to a 408 amino acid protein with a calculated molecular weight of 45,808 Da. Transcript variants 3 and 4 differ in the 5′ and 3′ untranslated region and are synthesized from an alternative downstream translation start codon, leading to the same and shortest protein isoform C. Isoform C is lacking the first 141 amino acids, consisting of only 275 amino acids with a calculated molecular weight of 31,168 Da. In contrast to human *NCEH1*, the murine *Nceh1* gene transcribes into a single mRNA that encodes for a 408 amino acid protein with a calculated molecular weight of 45,739 Da (Figure 2A,B).

Orthologs of the human *NCEH1* gene are well conserved in 306 organisms, including mouse, rat, rhesus monkey, chimpanzee, pig, cattle, dog, and others (Table 1). All listed orthologs share between 82 and 98% sequence identity with the longest human transcript variant 1 of *NCEH1* and 85 to 98% identity with the isoform A on protein level. Orthologous genes give rise to proteins with amino acid sequence lengths of either 408 or 440, as compared to that of human isoform A that comprises 416 amino acids (Table 1).

## 3. Tissue Expression Pattern and Regulation of Expression

The tissue expression pattern of murine KIAA1363, as assessed by Northern blotting, showed a relatively high expression level in kidney followed by lower expression levels in heart, adrenal gland, and the least in brain. Virtually no signals of the hybridized probe were observed in liver and the adipose tissue [9]. Consistent with very low or virtually no detectable mRNA expression in adipose tissue, murine KIAA1363 mRNA expression was undetectable in differentiated 3T3-L1 adipocytes [9]. Furthermore, mRNA expression was also detectable in isolated mouse peritoneal macrophages and human-monocyte-derived macrophages [9].

Promoter sequences of the human and murine *NCEH1/Nceh1* genes that regulate its tissue-specific expression have not been investigated in detail. In any case, several studies have shown that KIAA1363′s mRNA is regulated by certain nuclear receptor [10], coactivators [11], and also by changes in dietary composition [12], demonstrating that KIAA1363 is not constitutively expressed but underlies regulation. Furthermore, the observed nutritional regulation implies that KIAA1363′s mRNA may also, similar to AADAC [3], be diurnally regulated; however, evidence for this is lacking. A recent study from Matsuoka et al. reported that mRNA expression of human KIAA1363 is regulated by retinoid-related orphan receptor alpha (RORα) [10]. In mammals, two putative response elements for RORα were found in the promotor region upstream of the transcriptional start codon of the human *NCEH1* gene (ROR response element 1, −1451/−1440; and ROR response element 2, −132/−121). In cell experiments using the human monocyte/macrophages cell line THP-1, phorbol-ester induced differentiation of THP-1 cells into macrophages induced expression of KIAA1363 and concomitantly of RORα. Furthermore, treatment of THP-1 cells with the SR1078 synthetic agonist of RORα/γ induced the expression of KIAA1363 at the mRNA, as well as protein level. Conversely, siRNA-mediated knockdown of RORα in the human hepatoma cell line HepG2 led to the downregulation of KIAA1363 mRNA expression. Thus, these results suggest that KIAA1363 is a direct target of RORα [10]. Endogenous ligands for RORα are suggested to be oxygenated sterols such as 7-oxygenated sterols and 24-hydroxycholesterol, indicating that RORα may serve as a sensor for oxysterols whose levels are regulated by cholesterol metabolism [13].

In neural tissue, murine KIAA1363 mRNA expression was reported to be regulated by the transcriptional co-activator PPARγ co-activator 1α (PGC-1α), which is considered as a master regulator of lipid and energy metabolism in central and peripheral tissues [14]. KIAA1363 transcripts were shown to be decreased in the anterior cortex of PGC-1α-ko mice and elevated in mice with an inducible overexpression of PGC-1α [11], demonstrating that KIAA1363 transcription is induced by the expression of PGC-1α in brain. Since PGC-1α is a co-activator of a number of nuclear receptors, including PPARs, liver X receptors, and farnesoid X receptor, as well as glucocorticoid receptor or thyroid hormone receptor, this could point toward a role of KIAA1363 in lipid and energy metabolism, as well as lipid/sterol biosynthesis and secretion.

Moreover, KIAA1363 mRNA expression was induced in a concentration-dependent manner upon chlorpyrifos oxon treatment of human breast cancer cells, which correlated with increased cell proliferation [15].

The fact that *KIAA1363* is annotated as *NCEH1* and another gene, namely *CES1*, is annotated as *NCEH*, has led to some confusion in the past, that *NCEH1* expression may be modulated by polyunsaturated fatty acids, insulin, or interleukin-33 [16,17,18]. However, original studies have reported these observations for the *NCEH* and not for the *NCEH1* gene [16,17,18].

More recently, the expression level of KIAA1363 in liver has been questioned and re-investigated. It was reasoned that KIAA1363 may be expressed exclusively in certain liver cell types, such as the lipid storing hepatic stellate cells, which may explain the aforementioned lack of KIAA1363 mRNA expression in liver [9,19]. Accordingly, qPCR measurements showed that murine KIAA1363 is expressed in non-parenchymal hepatic stellate cells but not hepatocytes [12]. Since hepatic stellate cells comprise around 5–8% of all liver cells, this may explain why KIAA1363 expression is basically undetectable in whole liver by Northern or Western blotting [9,12]. Interestingly, murine KIAA1363 mRNA expression in hepatic stellate cells was found to be induced (4-fold) upon stellate cell activation [12], a process that occurs in vitro upon cultivation of isolated stellate cells. This suggests that KIAA1363 expression may also be induced upon liver injury in vivo, a process known to activate hepatic stellate cells, which then initiates the trans-differentiation into myofibroblast-like cells and the concomitant loss of their lipid stores, most prominently of vitamin A. Thus, KIAA1363 may play a role in the cellular remodeling of activated hepatic stellate cells. Moreover, hepatic mRNA transcripts of murine KIAA1363 correlated inversely with the dietary vitamin A content in mice [12]. Mice fed a vitamin-A-deficient diet for 9 weeks displayed elevated hepatic mRNA levels of KIAA1363, whereas mice fed a vitamin-A-excess diet for 3 weeks showed decreased hepatic mRNA levels, compared to mice fed a standard chow diet [12].

In line with mRNA expression, KIAA1363 protein is highly abundant in kidney, brain, lung, and heart, followed by lower expression in spinal cord and testis in mice [19]. In other studies, protein expression of murine KIAA1363 was also observed in the adrenal gland [20], whereas low or no expression of KIAA1363 was observed in white, as well as brown, adipose tissue and liver, respectively [9,19,21]. In liver, murine and human KIAA1363 protein expression, similar as observed at the mRNA level, is apparently limited to a certain liver cell type, the hepatic stellate cells, but is not present in parenchymal cells [12]. Moreover, KIAA1363 protein expression has also been demonstrated in murine peritoneal macrophages and differentiated mature human macrophages. Consistently, KIAA1363 expression was found in foamy macrophages of atherosclerotic plaques from apolipoprotein-E-deficient mice, as well as of human atherosclerotic lesions [9,21].

## 4. Protein Structure: KIAA1363 Harbors an α/β-Hydrolase Fold

KIAA1363 belongs to the GDXG family of serine hydrolases, containing an α/β-hydrolase fold and the active serine consensus motif GXSXG, with the active serine within the GDSAG sequence [22,23]. Homology remodeling based on the crystal structure of *Archaeoglobus fulgidus* esterase indicated that the catalytic triad of murine KIAA1363 consists of Ser191, Asp348, and His378, as well as the oxyanion hole comprising His113, Gly114, Gly115, and Gly116 [19]. Site-directed mutagenesis of the active serine 191 and glycine 114 inactivated the enzymatic activity against the artificial substrate para-nitrophenyl butyrate, thereby confirming the importance of respective amino acids for enzymatic activity [24].

**Figure 3:**
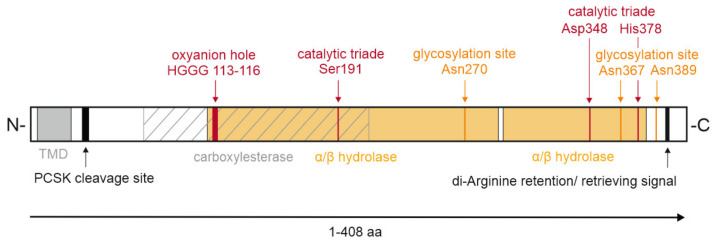
Domain organization of murine KIAA1363. Schematic representation of murine KIAA1363, with domain boundaries indicated. Single amino acids (aa) and sequences of specific functions are depicted. The α/β-hydrolase fold is colored orange. The area of the carboxylesterase domain is hatched. PCSK, pro-protein convertases subtilisin/kexin type serine protease; TMD, transmembrane domain.

Within the serine hydrolase superfamily, KIAA1363 belongs to the AADAC family. Amino acid sequence alignment with AADAC shows 44% identity and 63% semi-conserved residues (residues of the same type). Bioinformatical analysis showed a high phylogenetic kinship between KIAA1363 and HSL in distinct protein regions [9]. Protein sequence alignment of murine KIAA1363 with murine, rat, and human HSL demonstrated that the main structural homologies are found in the predicted α-helices and β-sheets, lipid binding, and catalytic domain containing the HG oxyanion motif and the active serine [9]. However, compared to HSL, KIAA1363 lacks the regulatory domain and the N-terminal region. Instead, KIAA1363 contains a putative N-terminal transmembrane domain consisting of a 23 amino acid–long hydrophobic stretch which facilitates sequestration at membranes. A further bioinformatic analysis using Eukaryotic Linear Motif scan or InterPro [25], analyses tools which classify protein sequences into families, functional domains, and conserved sites of proteins, confirmed the presence of an N-terminal transmembrane domain ranging from position 5 to 27 and predicted a di-arginine retention/retrieving signal ranging from position 394 to 397, possibly targeting KIAA1363 to the ER. Moreover, the Eukaryotic Linear Motif scan revealed an α/β-hydrolase domain ranging from position 109 to 290 and from 293 to 382, a carboxylesterase domain ranging from position 70 to 210, and a predicted pro-protein convertase subtilisin kexin cleavage site at position 32–36 for a potential proteolytic processing of KIAA1363 (Figure 3).

## 5. KIAA1363 Localizes to the ER

Based on the presence of a N-terminal transmembrane domain and the C-terminal di-arginine retention/retrieving signal in the amino acid sequence, KIAA1363 is predicted to localize to ER membranes. In cell fractionation experiments using cell lysates from mouse peritoneal macrophages and lysates of Expi293F^TM^ cells transiently expressing murine KIAA1363, its protein expression was found predominantly enriched in the microsomal fraction [9,12,21]. Immunostaining of endogenous KIAA1363 in mouse peritoneal macrophages, as well as expression of GFP-tagged murine KIAA1363 in RAW264.7 cells, confirmed ER-based localization [24]. To further delineate the role of the N-terminal region of murine KIAA1363 in ER targeting, Ishibashi and colleagues constructed a GFP-tagged KIAA1363 mutant that lacks the first 33 amino acids of the N-terminal region (ΔN-KIAA1363) [24]. While the wild-type GFP-tagged KIAA1363 showed localization at the ER in HEK293 cells, the ΔN-KIAA1363 mutant showed no localization at the ER [24]. Conversely, the generation of an GFP-tagged mutant only containing the 33 amino acid–long N-terminal region of KIAA1363 led to a similar ER localization, as observed for the full-length wild-type protein [24]. Based on these observations, the N-terminal region of KIAA1363 apparently serves as a membrane anchor targeting KIAA1363 to the ER.

Within the ER, the catalytic domain of KIAA1363 is facing the ER lumen: The orientation of murine KIAA1363 at the ER was investigated by the digestion of mouse peritoneal macrophages with the detergents saponin and digitonin [24]. In principle, the treatment of cells with digitonin only exposes cytosolic epitopes, while the treatment with saponin leads to the exposure of cytosolic and ER luminal epitopes. Permeabilization of mouse peritoneal macrophages with saponin but not with digitonin exposed KIAA1363 so that it was detectable by immunostaining, indicating that KIAA1363 is located within the ER lumen. Moreover, KIAA1363 was sensitive to proteinase K treatment in saponin-treated mouse macrophages, confirming that the soluble part of KIAA1363 is facing the lumen of the ER [24].

Furthermore, the same study also investigated targeting of KIAA1363 to cytosolic lipid droplets. However, cholesterol loading of mouse peritoneal macrophages by incubation with acetylated low-density lipoprotein did not result in a shift of endogenously expressed KIAA1363 from the ER onto lipid droplets, as assessed by immunostaining [24]. Similar results were obtained in RAW264.7 cells containing GFP-tagged or myc-tagged murine KIAA1363. The vast majority of KIAA1363 did not colocalize with BODIPY493/503-positive lipid droplets, even after cholesterol loading [24].

## 6. Post-Translational Modification: KIAA1363 Is Glycosylated

A Western blot analysis of murine KIAA1363 resulted in a double band at around 45 and 50 kDa [9,12]. Both bands have been shown to be a product of heterogenous glycosylation of KIAA1363. Treatment of HEK293 cells expressing murine KIAA1363 with the N-glycosylation inhibitor tunicamycin, as well as endoglycosidase digestion with peptide-N-glycosidase of membrane fractions derived from murine peritoneal macrophages, converted the KIAA1363-specific double band into a single 40 kDa band. Similar results were obtained by endoglycosidase H digestion of mouse peritoneal macrophage-derived membrane fractions, indicating that glycosylations are mainly formed at the ER, since Golgi-derived glycosylations are resistant to endoglycosidase H digest [24,26]. Bioinformatic analysis by Igarashi and colleagues showed that murine KIAA1363 possesses three potential glycosylation sites: Asn270, Asn367, and Asn389. Site-directed mutagenesis of the respective glycosylation sites, exchanging asparagine by glutamine, indicated that all three glycosylation sites are required for full glycosylation.

Similar to the murine protein, Western blot analyses of human KIAA1363 showed bands migrating at different heights, such as double bands at around 40 and 45 kDa in human THP-1 macrophages and at around 48 kDa in human primary hepatic stellate cells [9,12]. Calculated molecular weights of human KIAA1363 isoforms suggest the presence of isoform A and/or B with a calculated molecular weight of around 46 kDa in the respective cells. However, whether the lower band around 40 kDa reflects a cleavage form or whether it represents a glycosylated form of isoform C (calculated molecular weight of 31 kDa) is unknown.

So far, no other post-translational modifications have been reported for KIAA1363. However, bioinformatic analysis using NetPhos3.1, a prediction tool with an algorithm from neuronal network for (serine, threonine, or tyrosine) phosphorylation sites [27], revealed unspecific phosphorylation sites at positions 77, 98, 125, 295, 363, and 399 (with a score above 0.9); a protein kinase A phosphorylation site at position 4 (with a score above 0.8); and potential protein kinase C phosphorylation sites at positions 68, 76, 209, and 294 (with a score above 0.8). A further analysis with Eukaryotic Linear Motif scan [28] predicted a protein kinase A phosphorylation site at position 396–402. However, the literature does not provide evidence that KIAA1363 is a target of protein kinase A. In fact, in cell experiments, activation of protein kinase A by forskolin treatment of HEK293 cells expressing Flag-tagged KIAA1363 and its cultivation in media containing [^32^P]-labeled H_3_PO_4_ did not lead to the transfer of the radiolabel, and, thus, KIAA1363 was not phosphorylated under these conditions [24]. Moreover, also cAMP, known to induce protein kinase A activity [29], did not increase enzymatic activity of KIAA1363, suggesting that KIAA1363′s enzymatic activity is not induced under conditions where protein kinase A is activated [24].

## 7. The Enzymatic Activity of KIAA1363 Depends on Glycosylation

Glycosylations of KIAA1363 have been shown to be crucial for its enzymatic activity [24]. Three N-linked glycosylation sites, Asn270, Asn367, Asn389, were individually mutated to glutamine to prevent glycosylation but to retain characteristics such as polarity or charge on this amino acid residue. The exchange of Asn to Gln at the position 270, which is closest to the active site serine, led to a complete loss of the hydrolytic activity of murine KIAA1363 against the artificial substrate para-nitrophenyl butyrate. A 40% reduction of the hydrolytic activity was observed in the Asn367Gln mutant. Hydrolytic enzymatic activity of the Asn389Gln mutant was unaffected [24]. Interestingly, no changes in the cellular localization of these Asn to Gln mutants were observed. Thus, reduced enzymatic activity is independent of KIAA1363’s membrane association and might be due to changes in protein folding, since it is known that N-linked glycosylation is required for the correct folding of many glycoproteins [30].

## 8. The Multifunctional Roles of KIAA1363

### 8.1. Role of KIAA1363 in Detoxification of Organophosphates in Brain

**Figure 4:**
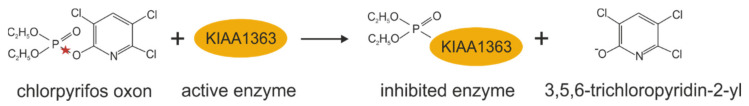
Chlorpyrifos oxon metabolization catalyzed by KIAA1363. Red stars indicate the bond that is hydrolyzed.

KIAA1363 has been shown to be involved in the detoxification of organophosphorus nerve poisons by metabolizing low levels of several insecticide metabolites in the brain (see Figure 4) [23,31]. Organophosphates are a major class of insecticides commonly used in agriculture, and, thus, human exposure to these compounds is widespread. The primary mechanism of the toxicity of organophosphates is due to the in vivo generation of several oxons, which inhibit the activity of organophosphate-sensitive serine hydrolases in the brain, such as acetylcholine-esterase, leading to a cholinergic overload [32]. In 2005, Cravatt and coworkers identified KIAA1363 as a chlorpyrifos oxon-binding protein by incubation of mouse brain membranes with ^3^H-labeled chlorpyrifos oxon in filtration assays [23]. They showed in their in vitro studies that the reaction of chlorpyrifos oxon with KIAA1363 leads to its rapid diethylphosphorylation, thereby hydrolyzing chlorpyrifos oxon and preventing the inhibition of a large number of organophosphate-sensitive brain enzymes. They showed that KIAA1363 is the principal chlorpyrifos oxon-binding protein in brain, since ^3^H-labeled chlorpyrifos oxon-labeling, -hydrolysis, and -metabolization was remarkably lower in brain membrane preparations of KIAA1363-ko mice than wild-type littermates (see also Table 2) [23]. Follow-up studies revealed that the genetic deletion of KIAA1363 in mice also attenuated in vitro chlorpyrifos oxon-hydrolysis in heart, kidney, lung, and testis membrane fractions and homogenates, as well as in spinal cord and muscle homogenates [19]. Intraperitoneal administration of chlorpyrifos led to increased toxicity in KIAA1363-ko mice, as is evident by markedly increased tremoring, acetylcholine-esterase inhibition, and mortality after 48 h compared to control mice, which exhibited only mild symptoms [33]. More severe toxicity was observed when parathion was administered to KIAA1363-deficient mice. Compared to wild-type mice, KIAA1363-ko mice displayed dramatically increased tremoring and acute mortality already 30 min after administration (see also Table 2) [33]. Results of in vivo studies clearly demonstrated that KIAA1363-ko mice were more sensitive to chlorpyrifos than wild-type mice [33]. Based on these observations, the authors concluded that KIAA1363 acts as a sequestration protein/hydrolase in organophosphate detoxification in nerve tissue, thereby protecting other organophosphate-sensitive enzymes from inhibition.

### 8.2. KIAA1363 Affects Ether Lipid Metabolism

**Figure 5:**
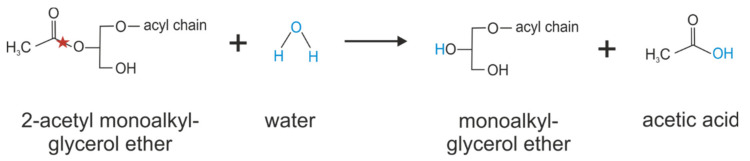
Catalyzing of 2-acetyl monoalkylglycerol ether hydrolysis by KIAA1363. Red stars indicate the bond that is hydrolyzed.

One year after (2006) the identification of KIAA1363 as organophosphate detoxifying enzyme in the brain, KIAA1363 was found in comparative metabolomic studies as 2-acetyl monoalkylglycerol ether (MAGE) hydrolase (see Figure 5) [36]. In that study, the global metabolite profile of the human ovarian cancer cell line SKOV-3 treated with the non-selective KIAA1363-inhibitor AS115 was compared with those of control cells and a reduction of the MAGE bearing C16:0 alkyl chain was observed. The same study then demonstrated that human KIAA1363 exhibits hydrolytic activity against 2-acetyl MAGE [36]. By hydrolyzing the metabolic intermediate 2-acetyl-MAGE, KIAA1363 affects cellular ether metabolism and the formation of lysophosphatidic acid and the phospholipid platelet-activating factor. In cell experiments using SKOV-3 cells, inactivation of KIAA1363 (by pharmacological means with AS115 or by shRNA-mediated silencing) correlated with reduced amounts of MAGE [36]. The reduction of MAGE levels in AS115- or shKIAA1363-treated SKOV-3 cells was accompanied by reduced alkyl-lysophosphatidyl-choline and alkyl-lysophosphatidic acid levels. The addition of [^13^C]-labeled MAGE to SKOV-3 cells led to the incorporation of [^13^C] in alkyl-lysophosphatidyl-choline and alkyl- lysophosphatidic acid, suggesting a direct pathway leading from MAGE to these lysophospholipids. Conversely, AS115-treated SKOV-3 cells showed reduced degradation of 2-acetyl MAGE that, in turn, led to an accumulation of phospholipid platelet-activating factor levels. Accordingly, the incubation of brain membrane preparations from KIAA1363-ko mice with 2-acetyl MAGE and CDP-choline led to elevated 2-acetyl MAGE, phospholipid platelet-activating factor, and lyso-phospholipid platelet-activating factor levels, demonstrating that 2-acetyl MAGE is the precursor in the biosynthesis of phospholipid platelet-activating factor [33,36].

Genetic deletion of KIAA1363 in mice led to the reduction of the majority of 2-acetyl MAGE hydrolase activity in brain, heart, lung, kidney, and testis, indicating that KIAA1363 is the predominant 2-acetyl MAGE hydrolase in these respective tissues (see also Table 2) [33,34]. No contribution of KIAA1363 to 2-acetyl MAGE hydrolase activity was reported in liver, skeletal muscle, and brown and white adipose tissue, respectively [33,34]. However, KIAA1363-ko mice did not show changes in brain MAGE levels, and MAGE levels of other tissues have not been reported (see also Table 2) [33]. Authors therefore suggested the existence of other 2-acetyl MAGE hydrolases which may compensate for the loss of KIAA1363 function [33]. 

Further studies conducted by Holly and colleagues described KIAA1363 as 2-acetyl MAGE hydrolase in human platelets (note: murine platelets do not express KIAA1363) [37]. They proposed that KIAA1363 regulates the accumulation of the ether lipid 2-acetyl MAGE, which affects protein kinase C signaling networks, which are crucial for human platelet activation in vitro and in vivo. They demonstrated that enzymatic inhibition of KIAA1363 by the specific inhibitor JW480 significantly reduced 2-acetyl MAGE hydrolase activity in human platelets that was accompanied with a decrease in C16:0, C18:0, and C18:1 MAGE levels [37]. Moreover, JW480-treatment of human platelets was associated with attenuated platelet aggregation in response to platelet-activating factors such as collagen, thrombin, and thromboxane A_2_ that impaired ex vivo thrombus growth [37]. The accumulation of cellular 2-acetyl MAGE ether lipids in JW480-treated human platelets led to the inhibition of protein kinase C activity, an essential signaling pathway, leading to the activation of the αIIbβ3 integrin, the most abundant surface receptor of platelets, mediating platelet activation [37]. Follow-up studies revealed that 2-acetyl MAGE was converted to its phosphorylated species upon KIAA1363 inactivation in human platelets. Phosphorylated 2-acetyl MAGE directly interacted with the lipid-binding domain C1α of two protein kinase C isoforms, which, in turn, inhibited protein kinase C activity in vitro, thereby affecting platelet activation. Thus, the authors concluded that phosphorylated 2-acetyl MAGE is the primary lipid involved in 2-acetyl MAGE-mediated regulation of human platelet activation [38]. The crucial role of KIAA1363 affecting circulating platelet function and thrombosis was assessed in several in vivo studies using rats [38]. Pharmacological inhibition of KIAA1363 by using JW480 protected rats against iron(III)chloride-induced thrombosis, as was apparent by the significantly increased occlusion time. Moreover, KIAA1363 inactivation impaired hemostasis in rats, as assessed by tail-bleeding assays [38].

### 8.3. KIAA1363 Affects Ether Lipid Metabolism in Cancer

The role of KIAA1363 in ether lipid metabolism may be most relevant in cancer cells since they exhibit elevated levels of ether lipids as compared to non-cancer cells [39,40,41]. KIAA1363 was originally characterized as a membrane-associated serine hydrolase highly upregulated in the invasive melanoma cell line MUM-2B, the breast carcinoma cell line MDA-MB-231, and in the ovarian carcinoma cell lines OVCAR-5 and SKOV-3 [22]. Many associations or Affymetrix GeneChip array studies found elevated KIAA1363 expression and activity in pancreatic cancer [42,43,44], breast cancer [22,45], gastric cancer [46], adrenal cortical carcinoma [47], colorectal cancer [48], and chronic myeloid leukemia [49]. Since KIAA1363 expression was consistently upregulated in invasive tumors, it may represent a biomarker for early diagnosis and a potential target for the treatment of different cancers [22,44,50]. Moreover, elevated KIAA1363 levels correlated with a poor prognosis and poor overall survival, thereby serving as a marker for malignant potential of the tumor [43,44,47]. Furthermore, several somatic mutations in the coding sequence of *NCEH1* have been identified (COSV56432875 [49], COSV56434458 [51], COSV56434234 [52], COSV56433597 [52], COSV56435557 [53], and COSV56432580 [54]) that are associated with tumor development in the lung, liver, intestine, endometrium, and nervous system. Chang et al. recently developed an activity-based fluorescent probe for KIAA1363, enabling direct visualization of KIAA1363 in an aggressive breast cancer xenograft model [45]. The use of the activity-based PET probe [^18^F]JW199 revealed heterogeneous abundance of KIAA1363 within tumors of breast and prostate cancer xenograft models. Elevated KIAA1363 abundance and activity was mainly observed at the growing edges of the tumors [45]. A similar distribution of KIAA1363 within tumors was confirmed by Western blot analysis and immunofluorescent imaging of whole xenograft tissue sections, showing increased KIAA1363 expression in tumor boundaries compared to tumor cores [45].

Mechanistically, it has been shown that KIAA1363 is the primary 2-acetyl MAGE hydrolase in tumor cells [36]. Moreover, shRNA-mediated silencing of KIAA1363 in SKOV-3 cells led to reduced MAGE, lysophosphatidyl-choline, and lysophosphatidic acid levels. The shKIAA1363 SKOV-3 cells showed impaired migration but not proliferation, as assessed in in vitro cell migration and proliferation assays. Remarkably, injection of shKIAA1363 SKOV-3 cells (exhibiting reduced 2-acetyl MAGE hydrolase activity) into immune-deficient mice led to a significantly reduced tumor growth rate compared to controls [36]. However, the addition of lysophosphatidic acid rescued the migratory activity of shKIAA1363 SKOV-3 cells [36]. Further studies showed that pharmacological inhibition of KIAA1363 by JW480 also completely abolished 2-acetyl MAGE hydrolase activity leading to reduced MAGE levels, impaired cell migration, invasion, and in vivo tumor growth of the prostate cancer cell line PC3 [55]. Based on these observations, the authors concluded that KIAA1363 serves as a critical node connecting ether lipid signaling and the production of pro-tumorigenic lipids such as alkyl-lysophosphatidic acid, thereby promoting cancer pathogenesis [36,55].

### 8.4. Role of KIAA1363 in Neutral Cholesterol Ester Hydrolysis

**Figure 6:**
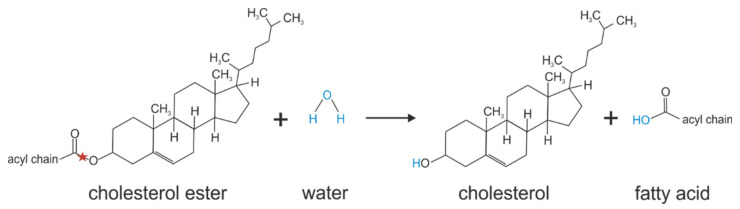
Cholesterol ester hydrolysis catalyzed by KIAA1363. Red stars indicate the bond that is hydrolyzed.

The multifunctional lipase HSL has been thought for some time to be responsible for cholesterol ester hydrolase activity in macrophages (see Figure 6) [56,57,58]. However, the observation that HSL-ko mice show almost normal levels of cholesterol ester hydrolase activity in macrophages questioned this assumption [59,60]. Two other enzymes, TGH/TGH-1 (Ces3 in mouse and CES1 in human) and TGH-2 (CesML1 in mouse), had been shown to exhibit cholesterol ester hydrolase activity, but in comparison to HSL at much lower levels [61,62]. Thus, Ishibashi and colleagues reasoned that another enzymes/neutral cholesterol ester hydrolase must be involved in neutral cholesterol ester hydrolysis in macrophages, thereby affecting foam cell formation and the development of atherosclerosis [9]. In a bioinformatic approach, screening a gene database for structural homologies against known lipases, they identified KIAA1363 as a possible candidate. They showed in functional studies that KIAA1363 exhibits hydrolase activity against cholesterol esters, using in vitro activity assays and HEK293 cell lysates expressing murine KIAA1363. Consequently, they renamed the enzyme as NCEH1 [9]. Moreover, they showed by adenovirus-mediated silencing of KIAA1363 in murine peritoneal macrophages and human monocyte-derived macrophages that KIAA1363 accounts for around half of the in vitro neutral cholesterol ester hydrolase activity [9,21]. Accordingly, KIAA1363-deficient murine peritoneal macrophages showed a reduction of microsomal neutral cholesterol ester hydrolase activity, whereas cytosolic activity was unaffected (see also Table 2) [35]. In cell experiments, the expression of KIAA1363 in macrophages also affected cellular cholesterol ester turnover: adenovirus-mediated expression of KIAA1363 in human THP-1 cells led to elevated in vitro cholesterol ester hydrolase activity and to decreased cellular cholesterol ester content after incubation with acetylated low-density lipoproteins, and to increased cholesterol efflux in the presence of high-density lipoproteins as an exogenous lipid acceptor [9,21]. Conversely, pharmacological inhibition of murine KIAA1363 with paraoxon (unspecific inhibitor that inhibits KIAA1363 at IC_50_ of 17 nM [31]), as well as the genetic deletion of KIAA1363 in acetylated low-density lipoprotein-loaden murine peritoneal macrophages, led to elevated cellular cholesterol ester levels and decreased cholesterol efflux [35,63]. Furthermore, prominent KIAA1363 expression was observed in atherosclerotic lesions of aortas from apolipoprotein E-ko mice, as well as in human atheromatous plaques [9,21]. The role of KIAA1363 in the development of atherosclerosis was assessed in in vivo studies, using KIAA1363-deficient apolipoprotein E-ko mice. When KIAA1363-ko mice were crossbred with apolipoprotein E-ko mice and fed a Western-type diet, areas of the aortic surface covered by atherosclerotic lesions were larger as compared to the control mice [35]. This accelerated the development of atherosclerosis in KIAA1363-deficient apolipoprotein E-ko mice and was associated with elevated free cholesterol and cholesterol ester levels in atherosclerotic lesions, as well as unchanged plasma cholesterol levels and an unchanged lipoprotein profile (see also Table 2) [35]. Similar observations were made in KIAA1363-deficient low-density lipoprotein receptor-ko mice [35]. Based on these observations, the authors concluded that KIAA1363 is crucial for cholesterol ester hydrolysis in macrophages, thereby affecting the development of atherosclerosis without affecting circulating cholesterol and triglyceride levels [35]. Besides reverse cholesterol transport, macrophage apoptosis is another important hallmark of atherosclerosis. Follow-up studies showed that KIAA1363-deficient thioglycolate-elicited peritoneal macrophages were highly prone to oxysterol-induced apoptosis [64]. Incubation of KIAA1363-deficient thioglycolate-elicited peritoneal macrophages with 25-hydroxycholesterol caused a massive microsomal accumulation of 25-hydroxycholesterol ester, due to its defective hydrolysis, leading to elevated ER stress signaling, such as increased CCAAT/enhancer-binding protein-homologous protein levels [64]. Moreover, 25-hydroxycholesterol-induced apoptosis in KIAA1363-deficient thioglycolate-elicited peritoneal macrophages was rescued by the addition of acetyl-CoA acetyltransferase 1 inhibitors, leading to reduced cholesterol esterification [64].

Furthermore, Ohta et al., reported an 18% reduction of in vitro cholesterol ester hydrolase activity in livers of KIAA1363-ko mice that, however, did not result in elevated hepatic cholesterol ester levels [20]. Interestingly, KIAA1363 deficiency provoked the enlargement of adrenal glands (by 12%) in mice. This enlargement was associated with a 30% accumulation of cholesterol ester but unchanged microsomal cholesterol ester hydrolase activity. A more pronounced adrenal gland enlargement and cholesterol ester accumulation was also observed in HSL-deficient mice (see also Table 2). Interestingly, the double knockout of both cholesterol ester hydrolases, HSL and KIAA1363, in mice showed an additive effect in adrenal gland enlargement that was associated with cholesterol ester accumulation. However, KIAA1363 deficiency did not provoke any changes in adrenal corticosterone production under basal or adrenocorticotropic hormone-stimulated conditions [20]. Recently, Raftopulos et al. reported that the inhibition of KIAA1363 by JW480 reduced in vitro cholesterol ester hydrolase activity in human prostate cancer–derived C4-2B cells by 75% and increased cellular cholesterol ester levels of lipoprotein-loaded cells, thereby reducing lipoprotein-mediated cell proliferation, suggesting that cholesterol availability and cholesteryl ester turnover may affect prostate cancer cell proliferation and aggressiveness [65]. In addition to mouse models and human studies, KIAA1363 has also been reported to modulate neuronal cholesterol metabolism in *Caenorhabditis elegans*, thereby affecting α-synuclein-induced neurotoxicity in dopaminergic neurons [66].

The role of KIAA1363 in cholesterol ester hydrolysis is somehow controversial, as another group has not observed cholesterol ester hydrolase activity of KIAA1363 in in vitro activity assays and no contribution of KIAA1363 to cholesterol ester hydrolysis in macrophages (see also Table 2) [34]. Furthermore, also no changes in cholesterol ester hydrolase activity and cholesterol ester levels were observed in the investigated tissues of KIAA1363-deficient mice, including white and brown adipose tissue, cardiac and skeletal muscle, liver, kidney, and brain [34]. The reasons for this discrepancy between the studies by Buchebner et al. [34] and that of Ishibashi and colleagues are unclear. However, it has to be noted that two KIAA1363-deficient mouse models have been generated with different gene targeting strategies: KIAA1363-deficient mice used by Ishibashi and colleagues were generated by the replacement of exon 4 of the *Nceh1* gene, which contains the active site of KIAA1363, with a neomycin resistance gene [35]. KIAA1363-deficient mice used by Kratky and colleagues that where identical to the ones used by the Cravatt lab lack exon 1 of the *Nceh1* gene, containing the translation start [34]. Theoretically, both mouse models could express non-functional protein truncations. The mouse model generated by Ishibashi’s lab does not express mRNA that can be detected by a KIAA1363-specific probe. Such information for the KIAA1363-ko mouse of the Cravatt’s lab is not available. Since that mouse model lacks exon 1, theoretically a 275 amino acid protein truncation variant harboring the catalytic site but missing the N-terminal transmembrane domain required for ER retention could be expressed. This 275 amino acid protein truncation would resemble isoform c of the human variants.

### 8.5. KIAA1363 Affects Cellular Retinyl Ester Turnover in Hepatic Stellate Cells

**Figure 7:**
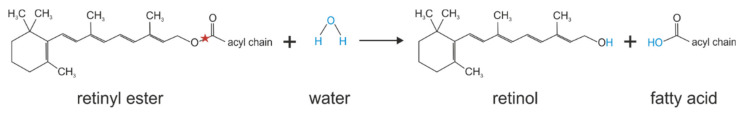
Retinyl ester hydrolysis catalyzed by KIAA1363. Red stars indicate the bond that is hydrolyzed.

In a recent study, Wagner et al. provided evidence that KIAA1363 also acts as retinyl ester hydrolase (see Figure 7) [12]. They found in in vitro activity assays that the murine and human orthologs of KIAA1363 exhibit hydrolase activity against retinyl palmitate, which was completely inhibited by the addition of the KIAA1363-specific inhibitor JW480 [12]. Furthermore, KIAA1363 accounted for the majority of in vitro retinyl ester hydrolase activity in primary murine and human hepatic stellate cells and affected cellular retinyl ester turnover in cell experiments [12]. While overexpression of murine KIAA1363 in COS-7 cells lowered cellular retinyl ester levels after retinol loading and serum-starvation, pharmacological inhibition and siRNA-mediated silencing of KIAA1363 partly or completely abolished retinyl ester degradation in serum-starved murine and human hepatic stellate cells, respectively [12]. Although in vitro studies and cell experiments point toward a role of KIAA1363 in retinoid metabolism, so far, any changes in retinoid homeostasis in KIAA1363-ko mice have not been reported. Obviously, KIAA1363-ko mice do not show decreased fertility, embryonic malformation, intestinal complications, dermatitis, immunity or vision deficits, or the like [67]. This may indicate that, under normo-physiological conditions, KIAA1363 does not induce any apparent changes in retinoid homeostasis. The observation that KIAA1363 expression is upregulated upon stellate cell activation may indicate that KIAA1363 is involved in the remodeling of activated stellate cells and may promote the loss of the vitamin A stores.

## 9. Conclusions

Given the diverse ester substrates that have been identified for KIAA1363, this enzyme obviously plays a multifunctional role under normo-physiological and pathological conditions. To date, the known substrates point toward functional involvement in the detoxification of organophosphates in the brain, in ether metabolism in cancer and platelets, in neutral cholesterol ester hydrolysis in macrophages, and in retinyl ester turnover in the liver. The diverse enzymatic activities of KIAA1363 raise the questions whether or not additional substrates for KIAA1363 exist that may be physiologically more relevant under normo-physiological conditions and, thus, may translate into apparent physiological changes in a KIAA1363-ko model. Such substrates may include, for instance, monoglycerides or lysophospholipid species, perhaps similar to AADAC, with shorter acyl moieties, that may be involved in lipid remodeling and thus may not impact overall lipid homeostasis.

The shared features of KIAA1363 with AADAC further suggest that, altogether, the members of the AADAC family may exhibit similar and overlapping substrate preferences and thus may, to some extent, compensate for each other. This view is bolstered by the conserved structural homologies of the catalytic domain of KIAA1363, as well as AADAC to that of HSL, suggesting that these enzymes may share a similar architecture and thus may exhibit conserved substrate preference. This hypothesis has been tested in yeast, where KIAA1363 (AADACL1) was unable to rescue the cholesterol acetate accumulation phenotype of the AADAC yeast ortholog Say1Δ-mutant [6], suggesting that the two enzymes have different substrate specificity, with the latter lacking sterol acetate hydrolase activity. This experimental evidence that heterologous expression of KIAA1363 does not affect cellular sterol acetate hydrolase activity in yeast is in line with reports on murine KIAA1363 that it actually does not exhibit hydrolytic activity against cholesterol esters [41]. However, several other studies have shown the opposite, and, thus, this conflicting view on the functional role of KIAA1363 in cholesterol ester turnover is, at the moment, difficult to reconcile [9,34,35]. Irrespective of the functional role of KIAA1363 in cholesterol ester turnover, the observation that neither HSL deficiency nor KIAA1363 deficiency has any effect on the hydrolysis of preformed, endogenously contained cholesterol ester stores in mouse peritoneal macrophages [9,34,35], suggests the existence of (an)other, as yet unidentified CE hydrolase(s) in macrophages.

The notion that KIAA1363 may also play a functional role in vitamin A turnover implies that a knockout model could show apparent defects in health and development. However, given that mice under standard laboratory chow feeding receive sufficient amounts of dietary vitamin A for the maintenance of body’s vitamin A demands, it appears unlikely that a phenotype due to dysregulated vitamin A homeostasis would develop if mice were not challenged with a vitamin-A-deficient diet. The lack of health defects has also been reported, for instance, for the lecithin retinol acyltransferase–knockout mice (that lack hepatic retinyl ester store) when maintained on a vitamin-A-containing chow diet [68,69]. Future studies on unraveling the vitamin A phenotype of KIAA1363-ko mice may shed light onto the physiological role of this enzyme in retinoid metabolism. The fact that KIAA1363 is expressed in hepatic stellate cells and not in hepatocytes renders it unlikely that KIAA1363 accelerates the export of retinol via binding to its specific transport protein (RBP4) from the liver. However, since its expression is increased upon hepatic stellate cell activation, it suggests that KIAA1363 may indeed affect vitamin A homeostasis under pathological conditions such as liver fibrosis and may thereby promote the hydrolysis of retinyl ester and accelerate the loss of hepatic retinyl ester stores.

Strikingly, KIAA1363-ko mice do not show any gross phenotype. Thus, any physiological role of this enzyme cannot be easily derived from the knockout mouse model. This obviously raises the question of whether KIAA1363 is actually involved in the metabolism of other ester substrates that have been currently not investigated in the literature. Furthermore, this lack of phenotype could also point toward a role in fine-tuning the turnover of metabolites rather than playing a rate-limiting role in any of these. Thus, in this scenario, a striking metabolic change and, thus, a KIAA1363-dependent phenotype would only become apparent when that particular pathway is challenged such as under more pathological conditions of xenobiotic intoxication, cancer development, or artificial in vitro cell experiments.

At this point, it appears that KIAA1363 expression levels can serve as a prognostic marker in certain cancer cell types. Furthermore, KIAA1363′s functional role in ether lipid metabolism appears to be the underlying mechanism in line with induced cancer development. Further research is required to dissect the functional role of KIAA1363, particularly in neutral lipid ester metabolism, including retinyl esters.

## Figures and Tables

**Table 1:** KIAA1363 orthologs.

	mRNA			Protein		
Species	Accession	Identity to Human *NCEH1* Transcript Variant 1	CDS Length (bp ^1^)	Accession	Identity to Human KIAA1363 Variant Isoform A	Length (aa ^2^)
*Mus musculus*	NM_178772.3	82%	1227	NP_848887.1	86%	408
*Rattus norvegicus*	NM_001127524.2	82%	1227	NP_001120996	85%	408
*Macaca mulatta*	XM_001084048.4	96%	1323	XP_001084048	97%	440
*Pan troglodytes*	XM_526382.7	98%	1323	XP_526382.2	98%	440
*Sus scrofa*	NM_001243484.1	87%	1227	NP_001230413.1	86%	408
*Bos taurus*	NM_001123034.1	88%	1227	NP_001116506	91%	408
*Canis lupus familiaris*	XM_545295.7	86%	1227	XP_545295.3	91%	408

^1^ bp, base pairs; ^2^ aa, amino acids.

**Table 2:** Phenotypical changes in KIAA1363-ko mice.

Proposed Function	Phenotypical Change	Reference
organophosphate detoxification	60–90% reduced [^3^H]chlorpyrifos oxon-hydrolysis in KIAA1363-ko brain, heart, spinal cord, kidney, lung, muscle, and testis membrane fractions; chlorpyrifos injection markedly increased tremoring, acetylcholine-esterase inhibition, and 48 h mortality in KIAA1363-ko mice; parathion injection dramatically increased tremoring and acute mortality in KIAA1363-ko mice.	Nomura et al., 2005 [23] Nomura et al., 2006 [19] Nomura et al., 2008 [33]
2-acetyl monoalkylglycerol ether hydrolysis	25–95% reduced 2-acetyl monoalkylglycerol ether hydrolase activity in whole homogenates and membrane fractions of KIAA1363-ko brain, heart, lung, testis, and kidney, as well as in lysates of KIAA1363-deficient murine peritoneal macrophages; no differences in brain monoalkylglycerol ether levels.	Nomura et al., 2006 [19] Buchebner et al., 2010 [34]
neutral cholesterol ester hydrolysis	30–50% reduced neutral cholesterol ester hydrolase activity and impaired cellular cholesterol ester degradation in KIAA1363-deficient murine peritoneal macrophages; adrenal gland enlargement and elevated cholesterol ester levels in KIAA1363-ko mice; unchanged neutral cholesterol ester hydrolase activity and cholesterol ester levels in tissues of KIAA1363-ko mice; KIAA1363/apolipoprotein E and KIAA1363/low-density lipoprotein receptor double-ko mice show accelerated development of atherosclerosis.	Sekiya et al., 2009 [35] Buchebner et al., 2010 [34] Ohta et al., 2011 [20]
